# Pharmacological Differences between Native Homomeric Transient Receptor Potential Canonical Type 4 Channels and Heteromeric Transient Receptor Potential Canonical Type 1/4 Channels in Lateral Septal Neurons

**DOI:** 10.3390/ph16091291

**Published:** 2023-09-13

**Authors:** Kevin D. Phelan, U Thaung Shwe, Fang Zheng

**Affiliations:** 1 Department of Neurobiology and Developmental Sciences, University of Arkansas for Medical Sciences, Little Rock, AR 72205, USA; phelankevind@uams.edu; 2 Department of Pharmacology and Toxicology, University of Arkansas for Medical Sciences, Little Rock, AR 72205, USA

**Keywords:** TRPC, epileptiform bursting, mGluRs, ML204, La^3+^

## Abstract

Given the unique expression patterns and revelations of its critical involvement in a host of neurological disorders, the TRPC1/4/5 subgroup has become an intense target of drug development, and some compounds are now in clinical trials. However, little is known about the exact subunit composition of this subfamily of TRPC channels in various native tissues, and whether it has functional and pharmacological implications. In this study, we investigated the effects of two TRPC4 modulators located in the lateral septum, in which a metabotropic glutamate receptor (mGluR) agonist-induced plateau potential is mediated by TRPC channels composed of TRPC1 and TRPC4. Lateral septal neurons were recorded intracellularly in brain slices using sharp electrodes. Drugs were applied via bath superfusion. We showed that the plateau potential in mice lacking TRPC1 is modulated by ML204 and La^3+^ in a manner that is like homomeric TRPC4 channels in artificial expression systems. However, the plateau potential that is primarily mediated by heteromeric TRPC1/4 channels in lateral septal neurons in wildtype mice was modulated differently by ML204 and La^3+^. Our data suggest that native homomeric TRPC4 channels and heteromeric TRPC1/4 channels are pharmacologically distinct, and the current drug development strategy regarding TRPC1/4/5 may need to be reevaluated.

## 1. Introduction

The canonical transient receptor potential channel (TRPC) family belongs to the mammalian *trp* superfamily of non-selective cation channels [1,2,3]. Mammalian TRPC channels are homologues of drosophila *trp* channels, which are calcium-permeable phosphoinositide-sensitive cation channels activated by light via rhodopsin [4,5,6]. Unlike other family branches of mammalian *trp* superfamily which mediate various types of sensory function, TRPC channels are primarily activated by G-protein coupled receptors via the phospholipase C signaling pathway. TRPC-deficient mouse models have provided a host of information regarding the functional roles of TRPC channels and their involvement in diseases [7,8,9,10,11,12]. For example, TRPC4 channels are critical for the regulation of vascular function by endothelial cells. TRPC6 channels are involved in smooth muscle function in vasculatures, and TRPC6 mutations have been linked to human kidney diseases. Heteromeric TRPC1/4 channels play a role in epileptiform burst firing and excitotoxicity. Interest in small molecules targeting TRPC channels has been continuously growing in the last decade [13,14,15,16].

There are seven members in the mammalian TRPC family, which are generally divided into three subgroups: TRPC1, 4, 5; TRPC3, 6, 7; and TRPC2 [17]. Each TRPC can form functional homomeric channels in artificial expression systems except for TRPC1. Generally, members within a subgroup can also form heteromeric channels. The subunit composition of native TRPC channels has been a subject of debate. Previous studies have suggested that there are native heteromeric TRPC channels formed by members of the TRPC3/6/7 subgroup or members of the TRPC1/4/5 subgroup [18,19,20]. There are also reports of possible heteromeric channels formed by TRPC3 and TRPC4, which are members of two distinct TRPC subgroups [21]. However, a recent study has revealed a clear distinction between the TRPC3/6/7 subgroup and the TRPC1/4/5 subgroup [22]. Heteromeric TRPC channels formed by members of the TRPC1/4/5 subgroup are highly prevalent, whereas members of the TRPC3/6/7 subgroup exist mainly as homomeric channels. More critically, little is known about the functional and pharmacological implications of the subunit compositions of TRPC channels.

For the subgroup of TRPC1, 4, and 5, it is generally accepted that TRPC1 is incapable of forming homomeric channels in native tissues, and it always forms functional heteromeric channels with TRPC4 and TRPC5 [23,24,25]. On the other hand, TRPC4 and TRPC5 can form homomeric channels. Biophysically, heteromeric TRPC1/4 channels exhibit a negative slope region near the resting membrane potential [26,27] that amplifies subthreshold membrane signaling like NMDA receptors and persistent Na^+^ channels [28]. In contrast, homomeric TRPC4 and TRPC5 show nonlinearity in their I–V relationship at positive holding potentials [29,30]. The implications of this biophysical difference between heteromeric TRPC1/4/5 channels and homomeric TRPC4 or TRPC5 channels are unexplored. It remains controversial whether homomeric TRPC4 or TRPC5 channels are functionally and pharmacologically distinct from heteromeric TRPC1/4/5 channels.

In the brain, TRPC1 is ubiquitously expressed in all tissues [31], whereas TRPC4 and TRPC5 are prominently expressed in distinct brain regions [8,29,32,33]. TRPC4 is highly expressed in the hippocampal CA1 region and the lateral septum. On the other hand, TRPC5 is highly expressed in the hippocampal CA3 region and the amygdala [33,34]. Previous studies have shown that heteromeric TRPC1/4 channels are primarily responsible for the plateau potential underlying epileptiform burst firing elicited by group I metabotropic glutamate receptor (mGluR) agonists [26]. This notion is supported by the observations that only TRPC1 knockout and TRPC4 knockout diminish epileptiform burst firing and the genetic ablation of TRPC3, 5, 6, and 7 has no detectable effects [11,26]. Intriguingly, the effects of TRPC1 knockout are distinct from TRPC4 knockout. Whereas epileptiform busting is totally absent in TRPC4 knockout or TRPC1/4 double knockout mice, a group of lateral septal neurons exhibit epileptiform burst firing elicited by mGluR agonists. The epileptiform bursting in these lateral septal neurons in mice lacking TRPC1 is presumably mediated by native homomeric TRPC4 channels, which provides an opportunity to explore the pharmacological properties of native homomeric TRPC4 channels. In this study, we compared the pharmacological characteristics of epileptiform burst firing in mice lacking TRPC1 and control mice, to determine whether there are pharmacological distinctions between native homomeric TPRC4 and heteromeric TRPC1/4 channels. Our results indicate that native homomeric TRPC4 channels are pharmacologically distinct from heteromeric TRPC1/4 channels. These distinctions may have broad implications for the current efforts to develop small molecule modulators of TRPC1, 4, and 5 for novel therapy.

## 2. Results

**Figure 1:**
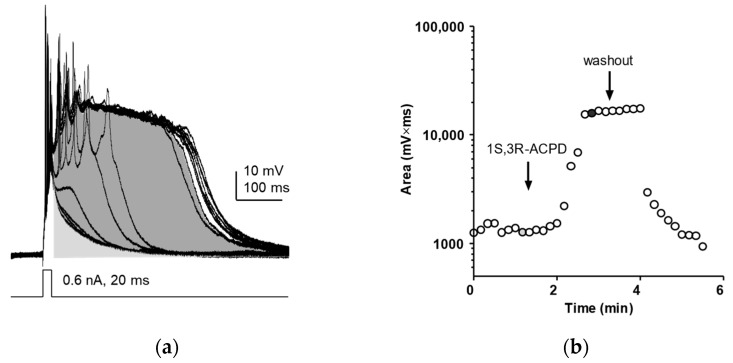
Quantitative comparison of the 1S,3R-ACPD-induced plateau potential in wildtype mouse lateral septal neurons. (**a**) Representative traces showing the normal decay of the membrane potential and the plateau potential induced by 30 µM 1S,3R-ACPD after spikes generated using a brief depolarizing current pulse. To quantify the 1S,3R-ACPD-induced plateau potential, the area under the curve (area; shaded) was measured from the end of the depolarizing current pulse to a time point after the end of the plateau potential (typically 1 or 2 s). (**b**) Changes in area elicited by the onset and washout of 1S,3R-ACPD were plotted. The solid symbol shown in (**b**) corresponds to the dark gray shaded trace shown in (**a**).

Lateral septal neurons were recorded intracellularly (see Material and Methods section). As reported previously [35], activation of mGluR1 elicits epileptiform bursts with an underlying plateau potential. Prior to drug application, each step potential was followed by a simple decay of the membrane potential back towards baseline, regardless of the presence of one or more spikes (Figure 1a). Bath perfusion of the mGluR1 agonist (1S,3R)-1-Aminocyclopentane-1,3-dicarboxylic acid (1S, 3R-ACPD) often resulted in membrane depolarization and spontaneous burst firing, and cells were then manually clamped to pre-drug holding potentials. In the presence of 1S,3R-ACPD, the same current step resulted in a stable plateau potential that lasted several hundred milliseconds (Figure 1a) to several seconds (in other neurons). The area under the curve (Area) was measured from the end of the current pulse to the end of the plateau potential (i.e., the time point of membrane potential returning to baseline; see Figure 1a) using Clampfit 10. The control area (light gray area, Figure 1a) was measured prior to bath perfusion of 1S,3R-ACPD for each neuron. The plateau potential was relatively stable and showed no signs of mGluR desensitization (Figure 1b). The plateau potential was completely reversible after washout of the 1S,3R-ACPD. After the 1S,3R-ACPD-induced plateau potentials were recorded, various TRPC channel modulators were co-applied via bath perfusion, and the changes in area were used to determine the effect of each TRPC channel modulator.

**Figure 2:**
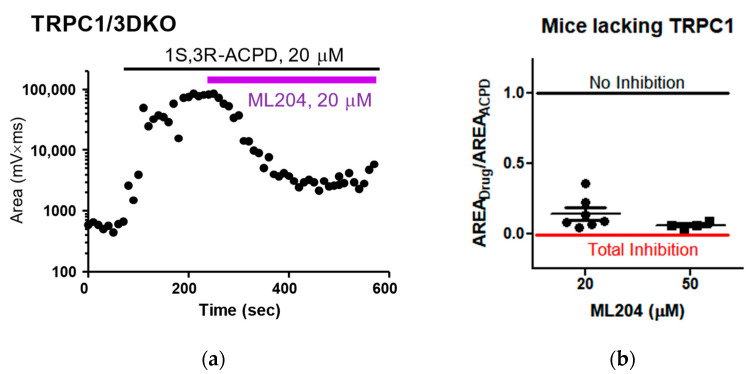
Inhibition of 1S,3R-ACPD-induced plateau potential by ML204 in lateral septal neurons in mice lacking TRPC1. (**a**) Changes in area elicited via bath superfusion of 1S,3R-ACPD (20 μM) and ML204 (20 μM) in a lateral septal neuron from TRPC1/3DKO mice were plotted. Note that ML204 attenuated the 1S,3R-ACPD-induced plateau potential. (**b**) The area of the plateau potential in the presence of a TRPC inhibitor divided by the area of the plateau potential in the presence of ACPD alone was used to quantify the efficacy of the inhibitor. Note that the 1S,3R-ACPD-induced plateau potential was greatly reduced by ML204 at 20 μM or 50 μM (*n* = 7, 4, respectively). The error bars represent mean ± S.E.M.

Since the plateau potential induced by 1S,3R-ACPD is entirely mediated by TRPC1 and TRPC4 [11], lateral septal neurons in mice lacking TRPC1 offered a unique opportunity to characterize homomeric TRPC4 channels pharmacologically. ML204 is the first selective TRPC4 inhibitor identified via high-throughput screening. Thus, we tested whether ML204 could attenuate ACPD-induced plateau potentials in mice lacking TRPC1 (TRPC1KO and TRPC1/3 double knockout (DKO)). We found that ML204 consistently attenuated 1S,3R-ACPD-induced plateau potentials (Figure 2a,b). Because the plateau potentials induced by 1S,3R-ACPD in mice lacking TRPC1 is presumably mediated by homomeric TPRC4 channels, our results indicate that ML204 is an effective inhibitor of native homomeric TRPC4 channels.

**Figure 3:**
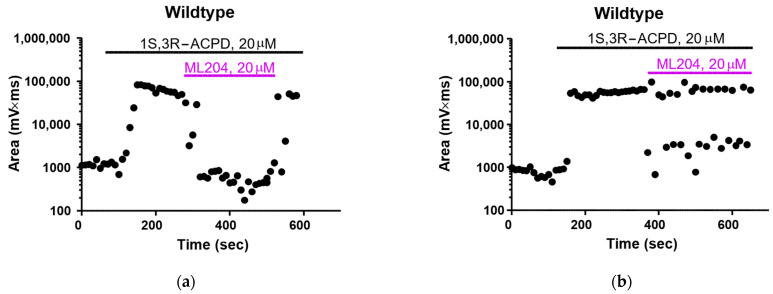
Distinct responses to ML204 in lateral septal neurons in wildtype mice: (**a**) a representative responder to ML204 (20 μM); (**b**) a representative non-responder to ML204 (20 μM).

**Figure 4:**
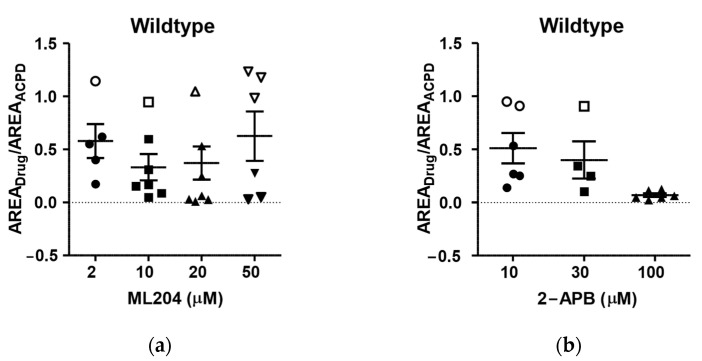
Distinct responses to ML204 in lateral septal neurons: (**a**) Lack of clear concentration dependency of the average ML204 inhibition (horizontal lines) of 1S,3R-ACPD-induced plateau. (**b**) Typical concentration dependency of 2-APB inhibition of 1S,3R-ACPD-induced plateau. Open symbols indicate non-responders at a given drug concentration whereas closed symbols indicate responders. Note that the average inhibition (horizontal line) is progressively greater at higher drug concentrations, and the variability is greatly diminished at the highest concentration. The error bars represent mean ± S.E.M.

The effect of ML204 on 1S,3R-ACPD-induced plateau potentials in wildtype mice was far more complex. We noticed that in some lateral neurons from the wildtype mice, the plateau potential induced by 1S,3R-ACPD (20 µM) was consistently attenuated by 20 µM ML204 (Figure 3a). This effect was completely reversible upon washout of ML204. In other neurons, ML204 failed to attenuate the plateau potential induced by 1S,3R-ACPD (Figure 3b). Instead, it caused a flicker in the 1S,3R-ACPD-induced response, i.e., an alternation between the appearance of full plateau potentials and smaller-amplitude depolarizing humps, as shown in the first few traces during the onset of the 1S,3R-ACPD-induced response shown in Figure 1a. Interestingly, ML204 was even less effective at 50 µM, and it had no detectable effects on the plateau potential induced by 1S,3R-ACPD in approximately half of the lateral septal neurons recorded from wildtype mice (open symbols, Figure 4a). In the other half of lateral septal neurons, ML204 at 50 µM caused a nearly complete block of the plateau potential induced by 1S,3R-ACPD (closed symbols, Figure 4a).

To better understand the complexity of its effects, we tested ML204 at lower concentrations (2 and 10 µM). Overall, there was little concentration dependency in the effects of ML204 on 1S,3R-ACPD-induced plateau potential in lateral septal neurons from wildtype mice (Figure 4a). The huge scatters of data persisted at all ML204 concentrations. In contrast, the effects of 2-Aminoethoxydiphenylborane (2-APB), a TRPC inhibitor, were more concentration-dependent (Figure 4b). Collectively, our data indicate that the plateau potentials in lateral septal neurons in wildtype mice are mediated by a mixture of TRPC channels with distinct pharmacological properties.

**Figure 5:**
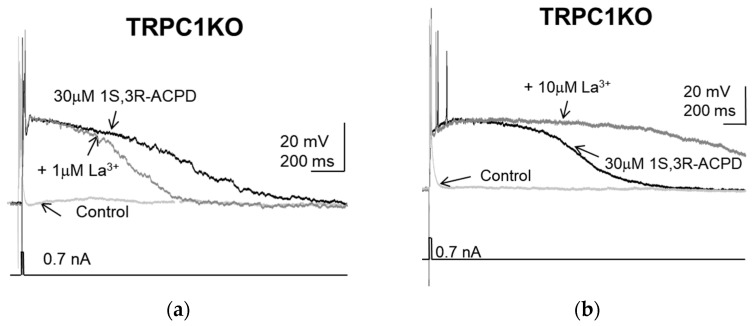
The effects of La^3+^ on 1S,3R-ACPD-induced plateau in lateral septal neurons in TRPC1KO mice: (**a**) Representative traces showing the inhibition of the plateau potential induced by 30 µM 1S,3R-ACPD by 1 µM La^3+^. (**b**) Representative traces showing the augmentation of the plateau potential induced by 30 µM 1S,3R-ACPD by 10 µM La^3+^.

In addition to ML204, another TRPC4 modulator that showed different effects in TRPC1KO mice and wildtype controls is lanthanum (La^3+^). La^3+^ was reported to exert dual effects on recombinant TRPC5 channels [36]. At 10 µM, La^3+^ potentiated currents mediated by TRPC5 channels, whereas at 100 µM, it inhibited TRPC5 channels. Since the amino acid residues mediating the potentiation by La^3+^ were conserved across TRPC5 and TRPC4, it was assumed that TRPC4 would also be potentiated by La^3+^ (Jung et al., 2003). However, a previous study showed that 1 µM La^3+^ inhibited the store-operated calcium current attributed to TRPC4 in endothelial cells [37]. To resolve these potentially conflicting reported effects of La^3+^, we attempted to determine the effects of La^3+^ at a wide range of concentrations. Indeed, the plateau induced by 1S,3R-ACPD in lateral septal neurons in TRPC1KO mice was reduced by 1 µM La^3+^ (Figure 5a). However, it was prolonged by 10 µM La^3+^ (Figure 5b). These observations suggest that the native homomeric TRPC4 channels that mediate the plateau potential are inhibited by 1 µM La^3+^ and potentiated by 10 µM La^3+^.

**Figure 6:**
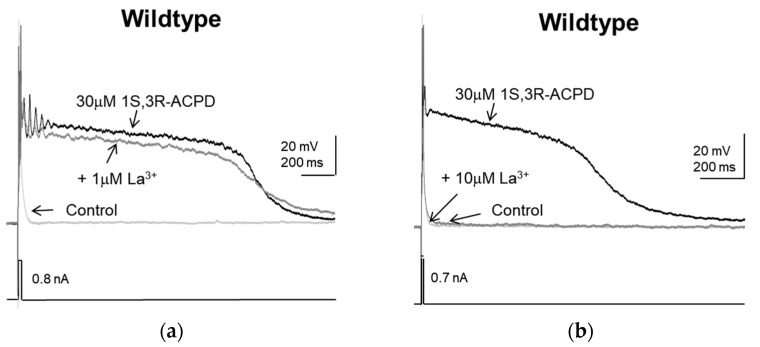
The effects of La^3+^ on 1S,3R-ACPD-induced plateau in lateral septal neurons in wildtype mice: (**a**) Representative traces showing the lack of inhibition of the plateau potential induced by 30 µM 1S,3R-ACPD by 1 µM La^3+^. (**b**) Representative traces showing the near total inhibition of the plateau potential induced by 30 µM 1S,3R-ACPD by 10 µM La^3+^.

In wildtype mice, the effects of La^3+^ on 1S,3R-ACPD-induced plateau in lateral septal neurons were different. At 1 µM, La^3+^ had negligible effects (Figure 6a). At 10 µM, La^3+^ completely blocked the plateau potential induced by 1S,3R-ACPD (Figure 6b). These observations indicated a need to compare the effects of La^3+^ within a broader concentration range. However, there was noticeable precipitation of La^3+^ in the bicarbonate-based artificial cerebrospinal fluid (ACSF) at 30 µM and higher concentrations. Since the exact concentration of La^3+^ in these solutions was unknown due to precipitation, we were unable to determine the effects of La^3+^ at concentrations higher than 10 µM.

**Figure 7:**
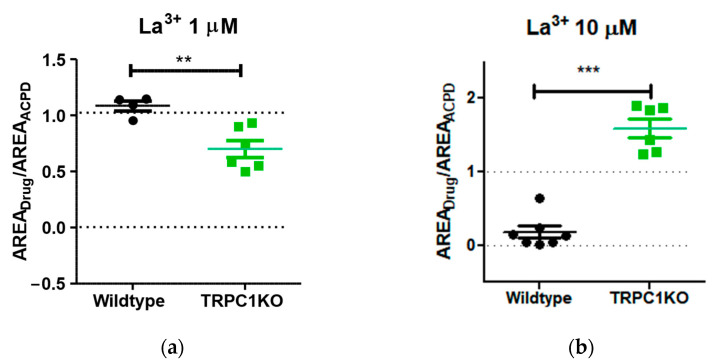
The effects of La^3+^ on 1S,3R-ACPD-induced plateau in lateral septal neurons in wildtype mice are significantly different: (**a**) At 1 µM, La^3+^ has negligible effects on the 1S,3R-ACPD-induced plateau in wildtype mice (*n* = 4), whereas it significantly inhibits the plateau in TRPC1KO mice (*n* = 6). **: *p* < 0.01, unpaired *t*-test. (**b**) At 1 µM, La^3+^ nearly abolishes the plateau in wildtype mice (*n* = 7), whereas it potentiates the plateau in TRPC1KO mice (*n* = 6). ***: *p* < 0.001, unpaired *t*-test. The error bars represent mean ± S.E.M.

Although our data were limited to the lower range of La^3+^ concentrations, the difference between TRPC1KO mice and wildtype control mice was highly significant at both concentrations (Figure 7). These results suggest that heteromeric TRPC1/4 channels that mediate the plateau potential in lateral septal neurons in wildtype mice are modulated differently from homomeric TRPC4 channels.

In summary, our results indicate that the plateau potential induced by mGluR agonists in wildtype mice and mice lacking TRPC1 exhibit distinct pharmacological properties. Since heteromeric TRPC1/4 channels are most prevalent in lateral septal neurons in wildtype mice, and homomeric TRPC4 channels are the only TRPC channels remaining in mice lacking TRPC1, the difference in responses to ML204 and La^3+^ between wildtype mice and mice lacking TRPC1 indicates that native homomeric TRPC4 channels are pharmacologically distinct from native heteromeric TRPC1/4 channels.

## 3. Discussion

The mGluR agonist-induced plateau potential in lateral septal neurons is mediated by TRPC channels with a defined subunit composition, i.e., TRPC1 and TRP4. Therefore, it provides a unique opportunity to characterize pharmacologically native TRPC channels composed of TRPC1 and TRPC4. Furthermore, mice lacking TRPC1 provide an opportunity to characterize the pharmacologically native homomeric TRPC4 channels. In the present study, we compared the effects of two TRPC4 modulators on the mGluR agonist-induced plateau potential in both wildtype mice which express primarily heteromeric TRPC1/4 channels and mice lacking TRPC1 which only express homomeric TRPC4 channels in the lateral septum. We found that the effects of ML204 and La^3+^ on the plateau potential in mice lacking TRPC1 are consistent with the expected effects of these modulators on homomeric TRPC4 channels. However, the effects of these two modulators on the plateau potential in wildtype mice are clearly distinct from what was observed in mice lacking TRPC1. These observations indicate that native homomeric TRPC4 channels are pharmacologically distinct from native heteromeric TRPC1/4 channels.

ML204 is a prototypical TRPC4 channel blocker identified via the high-throughput screening of recombinant homomeric TRPC4 channels [38]. However, a later study showed that mGluR agonist-induced plateau potential is poorly inhibited by ML204 [39]. Our data in this study revealed a high variability of ML204’s effects on plateau potential in wildtype mice. Intriguingly, a subgroup of lateral septal neurons in WT mice were totally insensitive to a high concentration of ML204, whereas the same concentration of ML204 completely blocked the plateau potential on the other subgroup.

The complexity of ML204’s effects could have potentially stemmed from the diversity of cellular electrophysical properties. Three types of lateral septal neurons with distinct electrophysiological properties have been described in rats [40] and mice (unpublished data). However, 1S,3R-ACPD induces epileptiform discharges with a plateau potential in all three cell types. Furthermore, there is no clear correlation between cell types and the responses to ML204. For these reasons, the observed differences in response to ML204 are unlikely to be related to diverse cellular electrophysiological properties.

Another possibility that could account for the existence of responders and non-responders to ML204 is that they might be mediated by distinct signaling complexes, leading to the activation of TRPC1/4 channels. If this is the case, one would expect to observe similar diversity in responses to drugs that inhibit the upstream signaling cascade. Our preliminary data show that U73122, a drug that blocks phospholipase C, consistently inhibits 1S,3R-ACPD-induced plateau potentials. We previously published that drugs that block mGluR1 consistently inhibit 1S3R-ACPD-induced plateau potential [26]. Therefore, the likelihood that the distinct responses to ML204 are caused by differences in upstream signaling is low.

The most plausible interpretation of our data regarding ML204 is that the ML-204-sensitive plateau potential in the subgroup of lateral septal neurons in WT mice is mediated by homomeric TRPC4 channels, whereas ML-204-insensitive plateau potential in the subgroup of lateral septal neurons in WT mice is predominantly mediated by heteromeric TRPC1/4 channels. Alternatively, the ML204-sensitive and ML204-insensitive TRPC channels in lateral septal neurons from wildtype mice may be heteromeric TRPC1/4 channels comprising different splice variants of TRPC4. A previous study reported that the longer TRPC4 variant contained an inhibitory domain that prevented efficient coupling to G-protein coupled receptors [41]. Thus, it is possible that the ML204-sensitive plateau potential in lateral septal neurons from wildtype mice could be heteromeric TRPC channels comprising TRPC1 and the longer splice variant of TRPC4. Further studies are needed to draw firm conclusions.

An important and intriguing question is how the introduction of TRPC1 into heteromeric TRPC1/4 channels alters their pharmacological properties. The structures of homomeric TRPC4 and homomeric TRPC5 channels have been resolved due to the rapid advancement in electron cryo-microscopy [42,43,44,45,46]. These structures have revealed multiple modulatory sites for channel gating [15]. Xanthine-derived compounds such as pico-145 share the same site with lipid activators of TRPC4/5 and inhibit both homomeric TRPC4 or TRPC5 and heteromeric TRPC1/4/5 channels. It is possible that ML204 binds to a modulatory site on the voltage sensor-like domain of TRPC4/5. La^3+^ binds to an aspartate residue in the outer vestibule of the TRPC4/5 channel [36]. A homomeric TRPC4 channel would have four aspartate residues that bind to La^3+^ [36], whereas a heteromeric TRPC1/4 channel would have fewer, depending on its stoichiometry. However, these structural insights have not yet been translated into a precise understanding of the gating of TRPC channels. Clearly, further studies are much needed to understand the pharmacological distinctions between homomeric TRPC4 channels and heteromeric TRPC1/4 channels suggested by our results.

Knowing whether heteromeric TRPC1/4/5 channels are functionally and pharmacologically distinct from homomeric TRPC4 or TRPC5 channels has huge implications for the future development of small molecule modulators of TRPC1/4/5 as a new therapy for human diseases. Previous studies have revealed the critical involvement of TRPC4 and TRPC5 in a host of neurological disorders [11,12,13,14]. It has been commonly assumed that heteromeric TRPC1/4/5 channels are responsible for these neurological disorders. It also has been commonly assumed that small molecule modulators identified via high-throughput screening using recombinant TRPC4 or TRPC5 will provide useful lead compounds for heteromeric TRPC1/4/5 channels. Our results raise questions about both assumptions. If heteromeric TRPC channels such as TRPC1/4/5 channels are functionally and pharmacologically distinct from homomeric TRPC4 or TRPC5 channels, the current drug development strategy regarding the TRPC1, 4, 5 subgroup may need to be reevaluated.

## 4. Materials and Methods

**Animals:** Adult mice (2–5 months old) were used in this study. Both males and females were used and pooled together as a single treatment group for statistical analysis. The animal study protocol was approved by the Institutional Animal Care and Use Committee of University of Arkansas for Medical Sciences (AUP 3011, approved on 9 September 2009; AUP 3336, approved on 21 August 2012; AUP 3641, approved on 20 August 2015).

**Brain Slice Preparation**: Mouse forebrain coronal slices containing the lateral septum were obtained in a manner described previously [26]. In brief, adult mice were anesthetized with ketamine (80 mg/kg, I.M.) and rapidly decapitated. The brain was removed, hand-blocked in the coronal plane, and cut into 500 µm thick serial sections on a Vibraslice (WPI) in a modified ice-cold ACSF. The ACSF was continuously bubbled with 95% O_2_ and 5% CO_2_ to maintain its pH at 7.3 to 7.4. Slices were collected at room temperature in oxygenated ACSF and allowed to recover for at least one hour prior to recording. Individual slices were placed between two nylon meshes submerged in our recording chamber and superfused with oxygenated ACSF at a rate of 1–2 mL/min. The superfusate was heated immediately prior to entering the chamber to maintain an experimental recording chamber temperature of 32 ± 1 °C. The composition of the modified ACSF was (in mM) as follows: NaCl, 117; KCl, 4.7; NaH_2_PO_4_, 1.2; CaCl_2_, 2.5; NaHCO_3_, 25; MgCl_2_, 1.2; and glucose, 11.5. All chemicals were obtained from Sigma or Fisher Scientific.

**Intracellular Recording**: Lateral septal neurons were impaled with microelectrodes pulled from filamented capillary glass (standard wall, 1.0 mm outer diameter, Sutter Instrument Co.) on a Flaming Brown Micropipette Puller (Model P-87/PC) and filled with 3 M potassium acetate to a final tip resistance of 60–90 MΩ. Voltage signals and applied current were recorded with an Axon Axoclamp 2B amplifier (Molecular Devices). The amplified voltage and current responses were digitized using pClamp with a Digidata interface (Molecular Devices). The membrane potential was held at −78 mV through injecting a small hyperpolarizing current, and a depolarizing current pulse (20 ms) was delivered at a constant interval (10 secs) to trigger spikes. The effects of each TRPC channel modulator alone on membrane potential and firing patterns were tested in a separate set of experiments.

**Drugs**: ML204 was kindly provided by Dr. Craig Lindsley at Vanderbilt University. LaCl3 was purchased from Sigma. All other drugs were purchased from Tocris Bioscience. All drug solutions were prepared daily from frozen stock solutions.

## 5. Conclusions

In this study, we characterized the pharmacological properties of native TRPC channels composed of TRPC1 and TRPC4. Epileptiform discharges with an underlying plateau potential induced by the mGluR1 agonist 1S,3R-ACPD are exclusively mediated by channels composed of TRPC1 and TRPC4 in wildtype mice and exclusively mediated by homomeric TRPC4 channels in mice lacking TRPC1 expression. Taking advantage of this unique preparation, we tested the effects of two TRPC1/4 modulators, ML204 and La^3+^. We found that ML204 consistently inhibited the 1S,3R-ACPD-induced plateau potential in lateral septal neurons recorded from mice lacking TRPC1, whereas it failed to inhibit the 1S,3R-ACPD-induced plateau potential in a subgroup of lateral septal neurons recorded from wildtype mice. La^3+^ potentiated the 1S,3R-ACPD-induced plateau potential in lateral septal neurons recorded in mice lacking TRPC1, whereas it inhibited the 1S,3R-ACPD-induced plateau potential in lateral septal neurons recorded from wildtype mice. Our data suggest that homomeric TRPC4 channels and heteromeric TRPC1/4 channels are pharmacologically distinct.

## Data Availability

Data is contained within the article.

## Abbreviations

1S,3R-ACPD	(1S,3R)-1-Aminocyclopentane-1,3-dicarboxylic acid;
2-APB	2-Aminoethoxydiphenylborane;
ACSF	artificial cerebrospinal fluid;
mGluR	metabotropic glutamate receptor;
ML204	4-Methyl-2-(1-piperidinyl)quinoline;
TRPC	Transient receptor potential channel;
TRPC1KO	TRPC1 knockout;
TRPC1/3DKO	TRPC1/3 double knockout
